# Exploring the gene expression network involved in the heat stress response of a thermotolerant tomato genotype

**DOI:** 10.1186/s12864-024-10393-0

**Published:** 2024-05-23

**Authors:** Salvatore Graci, Riccardo Aiese Cigliano, Amalia Barone

**Affiliations:** 1 Department of Agricultural Sciences, University of Naples Federico II, Portici, Naples, Italy; 2 Sequentia Biotech SL, C/ del Dr. Trueta 179, Barcelona, 08005 Spain

**Keywords:** *Solanum lycopersicum*, High temperatures, Abiotic stress, Candidate genes, Heat shock proteins, Heat shock factors, GDSL esterase/lipase, Gene co-expression network

## Abstract

**Background:**

The increase in temperatures due to the current climate change dramatically affects crop cultivation, resulting in yield losses and altered fruit quality. Tomato is one of the most extensively grown and consumed horticultural products, and although it can withstand a wide range of climatic conditions, heat stress can affect plant growth and development specially on the reproductive stage, severely influencing the final yield. In the present work, the heat stress response mechanisms of one thermotolerant genotype (E42) were investigated by exploring its regulatory gene network. This was achieved through a promoter analysis based on the identification of the heat stress elements (HSEs) mapping in the promoters, combined with a gene co-expression network analysis aimed at identifying interactions among heat-related genes.

**Results:**

Results highlighted 82 genes presenting HSEs in the promoter and belonging to one of the 52 gene networks obtained by the GCN analysis; 61 of these also interact with heat shock factors (Hsfs). Finally, a list of 13 candidate genes including two Hsfs, nine heat shock proteins (Hsps) and two GDSL esterase/lipase (GELPs) were retrieved by focusing on those E42 genes exhibiting HSEs in the promoters, interacting with Hsfs and showing variants, compared to Heinz reference genome, with HIGH and/or MODERATE impact on the translated protein. Among these, the Gene Ontology annotation analysis evidenced that only LeHsp100 (Solyc02g088610) belongs to a network specifically involved in the response to heat stress.

**Conclusions:**

As a whole, the combination of bioinformatic analyses carried out on genomic and trascriptomic data available for tomato, together with polymorphisms detected in HS-related genes of the thermotolerant E42 allowed to determine a subset of candidate genes involved in the HS response in tomato. This study provides a novel approach in the investigation of abiotic stress response mechanisms and further studies will be conducted to validate the role of the highlighted genes.

**Supplementary Information:**

The online version contains supplementary material available at 10.1186/s12864-024-10393-0.

## Background

Heat stress (HS) due to climate change stands out as a primary threat adversely impacting world crop production [1]. In the global warming era, it is expected that temperatures will rise between 2 and 5 °C by the end of the 21st century [2] inducing serious damage on plant growth and development, thus resulting in dramatic yield losses [3]. Tomato (*Solanum lycopersicum* L.) is one of the most valuable horticultural crops globally. It is constantly challenged by a wide range of environmental stresses causing yield losses and fruit quality alteration, although its sensitivity varies among genotypes [4]. High temperature can cause enzyme degradation that can hamper PSII function, decrease electron transport rates, inhibit Rubisco activase and decrease chlorophyll content, cause abortion of the male gametophyte and altered pollen tube development, and lead to reduction in fruit set and final yield [5, 6]. In this scenario, the selection and constitution of tolerant tomato genotypes is crucial for mitigating the impact of climate change. However, the long time required for traditional plant breeding in genotype selection and breeding cycles represents the main limitation to a prompt response of plant breeders to the increasing demand for food production [7]. During the last years, high-throughput technologies based on omics sciences, such as genomic and transcriptomic, have emerged in response to these limitations. Different authors have investigated plant genomes to identify candidate genes in response to HS. Olivieri et al. [8] and Cappetta et al. [9] employed a Genotyping-By-Sequencing (GBS) approach to uncover Single Nucleotide Polymorphism (SNP) and Insertion and/or Deletion (InDel) variants among a group of genotypes, combining genotyping data with the ones obtained from the phenotypic evaluation of key-traits responsive to HS. Through genome-wide association studies, Bineau et al. [10] and Alsamir et al. [11] identified quantitative trait loci (QTLs) related to phenotypic traits such as flowering, fruit production, plant vigor, etc. Additionally, variants within the promoter regions could contribute to enhancing the regulation of HS-related genes, thereby improving plant thermotolerance. Indeed, the presence of specific HS binding site sequences on target genes promoters could promote the activity of heat stress transcriptional factors (Hsfs) that bind to these motifs, thus enhancing gene expression under unfavorable stress conditions [12, 13]. In this context, over the past decade, several authors have investigated the transcriptomic response of tomato plants to HS by RNA sequencing (RNA-seq), which has become the main tool for transcriptome-wide analysis of differential gene expression and gene co-expression networks (GCN) [14]. Differential gene expression analysis allowed to compare the transcriptome profile of plants exposed to two or more experimental conditions, thus allowing the identification of candidate genes [15], while GCN analysis is a popular biology method used to construct gene networks and detect the central players (i.e., hub genes) within modules, thereby highlighting interactions among clusters of genes in order to study regulatory pathways [16]. The combination of these two analyses can improve understanding of defense mechanisms activated in response to HS.

The goal of the present study was to enhance the knowledge of the HS response in a tomato genotype previously selected in our laboratory (E42) for its high and stable production under high temperatures [8]. This purpose was pursued by investigating genomic and transcriptomic resources available in our laboratory or retrieved from public data. In particular, the genomic analyses conducted in our laboratory by Graci et al. [17] evidenced a high number of polymorphic regions compared to Heinz tomato reference genome, regions putatively introgressed from the heat-tolerant wild ancestor *S. pimpinellifolium.* In addition, a subset of candidate genes was selected in these polymorphic regions, in some cases also colocalizing in QTLs for high temperature responses. In order to further understand the E42 response to HS, in the present study we investigated the regulation pathways of HS-related genes using bioinformatic tools. To achieve this, firstly two heat stress elements (HSEs) were searched across the whole E42 genome, focusing on those mapping in the promoters and reported to be involved in regulating the expression of HS target genes when binding with Hsfs [18, 19]. Moreover, to investigate the gene interactions among tomato genes, public transcriptomic data of RNA-seq experiments were retrieved from the NCBI database, and a GCN analysis was performed. The combination of the results obtained from both the genomic and transcriptomic analyses, and the integration with the findings of Graci et al. [17], allowed us to narrow to 13 the number of HS-related candidate genes mapping in E42 polymorphic regions introgressed from the thermotolerant wild species *S. pimpinellifolium*. As a whole, the selected genes exhibit HSE binding motifs in the promoter and interact with transcriptional factors (TFs) involved in the response to high temperatures, as evidenced from the GCN analysis. In addition, their polymorphisms respect to the reference genome of the cultivated tomato Heinz may alter the amino acid sequences and function of the translated proteins. All these conditions found simultaneously in the selected genes may influence the response of E42 to HS.

## Methods

### Data collection

Resequencing data of the E42 genotype already available at the Department of Agricultural Sciences of University of Naples Federico II [17] were used to investigate the presence and the distribution of binding motif sequences in the promoter regions of the genotype for the tomato response to HS. Moreover, in order to investigate how tomato genes interact with each other, publicly transcriptomic data of RNA-sequencing (RNA-seq) experiments obtained from different tomato tissues were retrieved from NCBI database. Specifically, three count matrices belonging to the GSE152620, GSE199011 and GSE148217 of Gene Expression Omnibus (GEO) projects were directly downloaded from the database, as these were already annotated on the same tomato genome version (Tomato Genome version SL4.0 and ITAG4.0, available at the Solgenomics Network, www.solgenomics.net). The GSE152620 project included 12 leaf samples, the GSE199011 project presented 12 fruit mesocarp tissues at the red ripe stage, the GSE148217 included 120 fruit pericarp and epidermal tissues of the blossom end halves. In addition, nine RNA-seq samples obtained from flower samples within the GSE163914 GEO project were entirely processed starting from the raw FASTQ files. Details about the samples belonging to the four GEO projects were reported in Additional file 1.

### Promoter binding motifs investigation

Raw FASTQ files of the E42 genotype were processed as reported by Graci et al. [17]. The resulted filtered Variant Calling Format file (VCF) was converted into a consensus FASTA file by using the consensus command of bcftools [20]. Finally, the Liftoff tool [21] was used to lift over the coordinates of the genes from the tomato genome annotation ITAG4.0 (available at the Solgenomics Network). The Positional Weight Matrix (PWM) files of binding motifs related to HS were retrieved from the Jaspar database (https://jaspar.genereg.net/). The scanMotifGenomeWide.pl script of HOMER [22] was used with default parameters and setting a threshold of 5 to find the binding motif sequences across the E42 genome. Focusing on the motifs mapping in the promoter regions, two approaches have been used in order to obtain a consensus: (I) a BED file with the coordinates of a region of 3,000 bp from the gene start site was generated from the tomato genome annotation ITAG4.0, and the intersectbed command of bedtools [23] was used to extract the motifs mapping in those regions; (II) the ChIPseeker R package of Bioconductor [24, 25] was used with default parameters to retrieve the nearest genes around the motifs and annotate the genomic region of genes, in order to select only the binding sequences mapping in the promoter regions. The ITAG4.0 tomato genome annotation was firstly converted from GFF to TXDB file by using the GenomicFeatures R package of Bioconductor [26]. The peakAnno command of ChIPseeker was used to annotate the binding motifs within the 3,000 bp promoter region from the gene start site. Lastly, a gene enrichment analysis for GO terms was performed with the topGO R package of Bioconductor [27], starting from the genome annotation retrieved from Pannzer2 [28] obtained by using the ITAG4.0 tomato genome annotation as input file.

### Read mapping and transcript quantification

Within the GSE163914 GEO project, the raw FASTQ data of nine samples (SRR13312158, SRR13312159, SRR13312160, SRR13312161, SRR13312162, SRR13312163, SRR13312164, SRR13312165 and SRR13312166) were downloaded by using the NCBI SRA Toolkit (https://trace.ncbi.nlm.nih.gov/Traces/sra/sra.cgi?view=software), quality evaluated, filtered and trimmed using FastQC and Trimmomatic v.0.39 [29, 30] (http://www.usadellab.org/cms/?page=trimmomatic), setting the parameters as follow: LEADING:20 TRAILING:20 HEADCROP:10 MINLEN:35. Single trimmed reads were aligned with the *Solanum lycopersicum* reference genome (Tomato Genome version SL4.0, available at the Solgenomics Network, www.solgenomics.net) using STAR [31] with default parameters. Quality control of the mapping was performed with the Qualimap tool [32]. Finally, the number of reads mapping for each gene (tomato genome annotation ITAG4.0) were calculate by using featureCounts [33] in order to obtain a count matrix.

### Gene co-expression network analysis

The four count matrices belonging to the GSE152620, GSE199011, GSE148217 and GSE163914 GEO projects were merged. Starting procedures included data normalization performed with the HTSFilter R package of Bioconductor [34] and Principal component analysis (PCA), aimed to validate the reproducibility of RNA-seq data across technical replicates and also to compare the global expression patterns between the different tissues and conditions. GCN analysis was performed with the BioNERO R package of Bioconductor [35]. Pre-processing steps included data transformation conducted with the vst function of the DESeq2 R package of Bioconductor [36] followed by the removal of the non-expressed genes with the remove_nonexp function of BioNERO, setting min_exp 5 and min_percentage_samples 0.15. The filtered and normalized expression data were then used to reconstruct a GCN. The SFT_fit function was used to identify the most suitable β power that makes the network satisfy the scale-free topology. The calculated β power was used by the exp2gcn function to infer the GCN. The edge list of each module was extracted and filtered for weak correlations by using the get_edge_list function. In addition, the hub genes were identified with the get_hubs_gcn function. A gene enrichment analysis for GO terms was performed for all the obtained modules with the topGO R package of Bioconductor [27]. Networks were finally visualized with the Cytoscape software platform [37].

## Results

### Promoter binding motifs investigation

In order to investigate the occurrence of HSEs in the promoter regions of the E42 genotype, the presence of AGAAnnTTCTRGA [18] and CGTTGACY [19] motifs was assessed. Results showed that the E42 genome presented 705,726 AGAAnnTTCTRGA and 778,739 CGTTGACY sequences (Table 1). These files were then filtered to keep only the binding motif sequences mapping in the promoter regions of 3,000 bp from the gene start site by using two different approaches. With the first one performed with the insersect command of bedtools, results evidenced that more that 31,000 AGAAnnTTCTRGA and around 34,000 CGTTGACY were found in the promoter regions of E42, while considering the second approach conducted with the ChIPseeker analysis, around 24,000 AGAAnnTTCTRGA and more than 26,000 CGTTGACY were identified (Table 1). Interestingly, more than 90% of both motifs map on chromosomes 2, 4, 7 and 10 (Additional file 2). In addition, the two motifs were similarly distributed in the various regions of the E42 genome, showing 10.3% of the binding sequences within 3,000 bp from the gene start site (Additional file 3). By comparing the two methods used, a consensus file containing the common binding sites sequences mapping in the promoter regions retrieved from both the Bedtools and ChIPseeker analysis was generated (Additional file 4). Interestingly, all the motifs retrieved from the ChIPseeker analysis were common to the ones obtained by using Bedtools except for one CGTTGACY binding motif (Table 1).

**Table 1:** Number of the AGAAnnTTCTRGA and CGTTGACY binding motifs mapping in the promoters of E42 genome compared to the ones mapping on the whole genome, obtained with Intersectbed and ChIPseeker analyses, and their consensus

Tool	Motifs position	AGAAnnTTCTRGA motifs (*n*)	CGTTGACY motifs (*n*)
scanMotifGenomeWide	genome	705,726	778,739
Intersectbed	promoters	31,156	33,951
ChIPseeker	promoters	24,132	26,781
Consensus	promoters	24,132	26,780

**Fig. 1:**
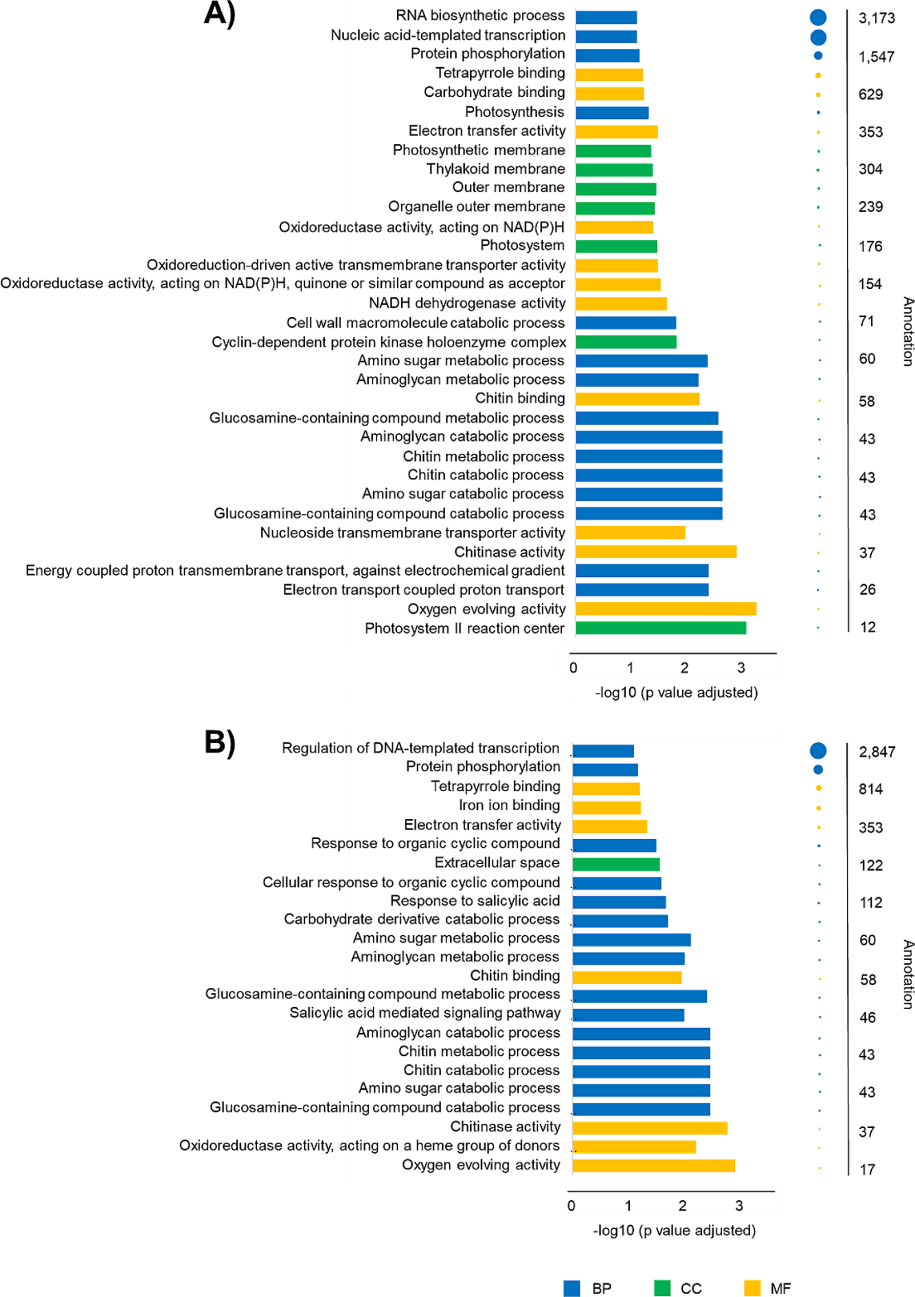
Plots of the GO enrichment analyses conducted on the E42 genes showing **(A)** AGAAnnTTCTRGA and **(B)** CGTTGACY binding motifs in the promoters

Considering the consensus file, a total of 11,032 genes were found to carry one or both the two binding motifs. Specifically, 9,472 genes showed AGAAnnTTCTRGA sequences and 9,708 genes presented CGTTGACY binding motifs in the promoters. In addition, the GO enrichment analysis was performed for the 11,032 genes showing the AGAAnnTTCTRGA and CGTTGACY binding motifs in the E42 promoters, respectively. As for the AGAAnnTTCTRGA binding site (Fig. 1A), in the biological processes (BP), the genes were mainly enriched in nucleic acid transcription and biosynthetic processes; in the cellular components (CC) they were mainly enriched in thylakoid and photosynthetic membranes, chitin and amino sugar metabolic processes, aminoglycan and amino sugar catabolic processes; while in the molecular function (MF) they were mainly enriched in oxygen evolving and oxidoreductase activities, NADH dehydrogenase activity, iron ion and carbohydrate binding, electron transfer activity. On the other hand, for the CGTTGACY binding site (Fig. 1B), in the BP the genes were mainly enriched in salicylic acid signaling and response, protein phosphorylation, regulation of DNA-template transcription; in the CC they were mainly enriched in glucosamine-containing compound and chitin metabolic processes, aminoglycan and amino sugar catabolic processes; while in the MF they were mainly enriched in oxygen evolving and oxidoreductase activities, iron ion and carbohydrate binding, electron transfer activity.

### Gene co-expression network analysis

**Fig. 2:**
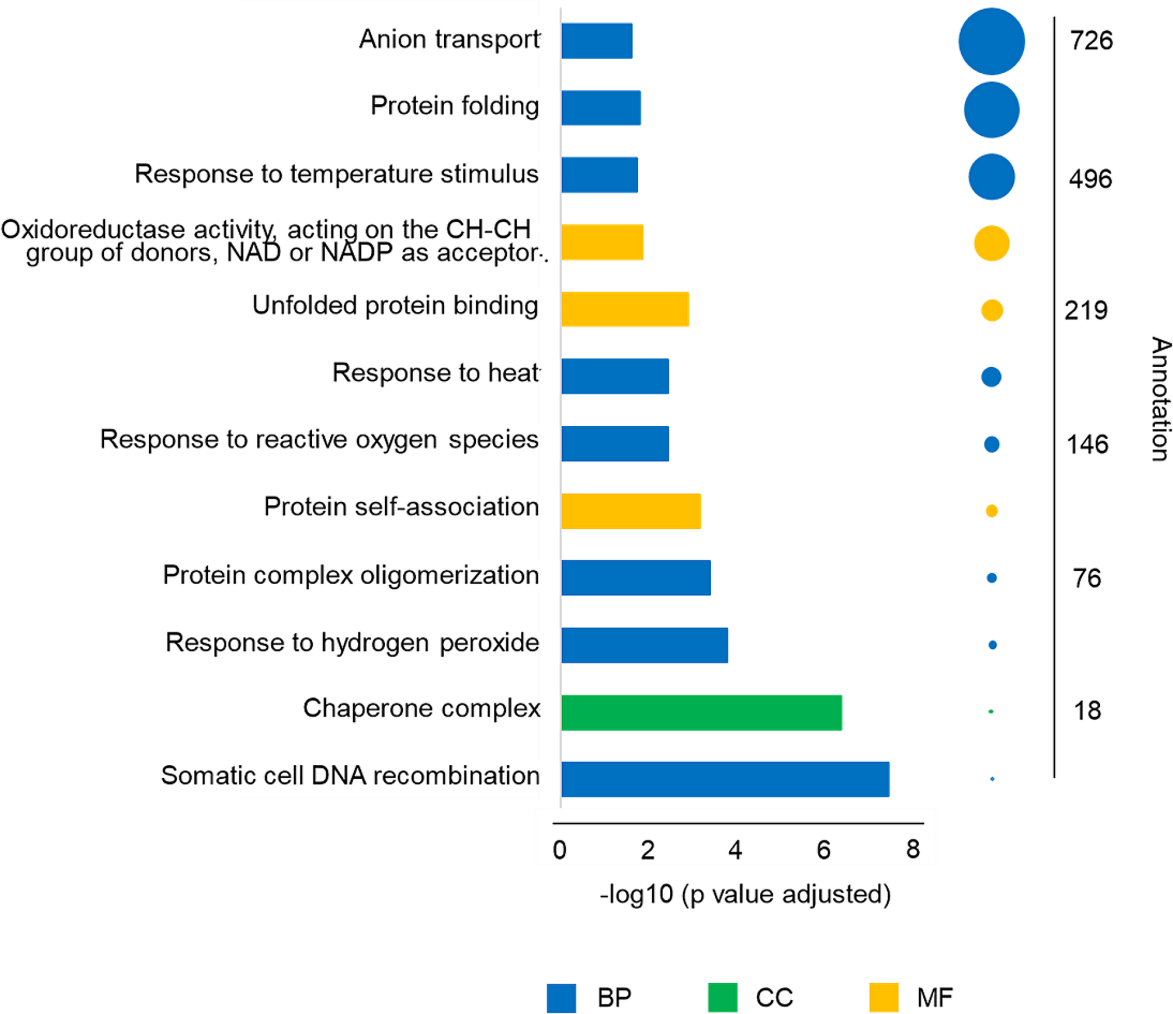
Plot of the GO enrichment analyses of the indianred2 module identified with the GCN analysis

In order to investigate the tomato network of genes involved in HS response and integrate this information with the genomic features of the E42 genotype, GCN analysis was performed on four count matrices of RNA-seq data. Among these, three were downloaded from three different GEO projects, while the fourth (belonging to the GSE163914 GEO project) was obtained by processing the raw reads. As a whole, these experiments included 153 tomato RNA samples sequenced from four tissues (leaves, flower buds, fruit mesocarp and fruit pericarp) and were analyzed to obtain a global view of all the gene interactions. The nine samples belonging to the GSE163914 GEO project were processed to obtain the matrix count (Additional file 5) As expected, most of the reads mapped in exonic regions. The four count matrices of RNA-seq data belonging to the four GEO projects were finally merged. Normalization of the count matrix allowed to identify the TMM value of 29.576 at the maximum Jaccard index of around 0.93 (Additional file 6). The TMM value was applied to remove all those genes whose expression values were lower than it. The PCA analysis showed that the four groups of tissues could be clearly distinguished, even if the fruit pericarp samples clustered in a group with a wider distribution probably due to the experimental conditions (Additional file 7). This dataset of 153 samples was used to perform the GCN analysis with the BioNero package. The pre-processing step allowed to obtain a dataset involving 21,276 expressed genes, while 12,779 genes were discarded as the expression values were lower than the threshold. The most suitable β power was 5 (Additional file 8) and when it was used to infer the GCN, 52 modules of genes were identified (Additional file 9). The honeydew module displayed the highest number of genes (5,262), followed by indianred2 (1,846) and cornsilk (1,648). In addition, 1,638 genes were identified as hubs with high correlation in the different modules (Additional file 10). The honeydew module showed the highest number of hub genes (474), followed by cornsilk (162), green4 (130) and indianred2 (127). Lastly, the GO enrichment analysis was carried out for all the 52 modules. Interestingly, the indianred2 module was shown to include the following GO terms related to the response to HS: response to temperature stimulus, response to reactive oxygen species, response to hydrogen peroxide, response to heat, protein folding for the BP term; chaperone complex for the CC term; unfolded protein binding for the MF term (Fig. 2).

### Selection of candidate genes for heat stress response

Results from HSE investigation and GCN analysis were finally merged, and 6,362 genes were identified, whose expression could influence the E42 thermotolerance. Among these, 25.4% of genes belong to the honeydew module and 8.9% to the indianred2 module, respectively (Additional file 11). This list was also combined with the list of 393 genes involved in the HS response in tomato, previously reported by Graci et al. [38], which included genes coding for TFs, Hsfs, Heat shock proteins (Hsps), flower-, pollen- and fruit set related genes. This combined analysis produced a list of 82 heat-related genes (Additional file 12) that contain at least one HSE binding site in the promoter and belong to one of the 52 networks resulting from the GCN analysis. The list of these genes include 43 Hsps also showing LeHsp100, 12 TFs, one flower-, 24 pollen- and 2 fruit set-related genes. Interestingly, 40% mapped on chromosome 2, 27% on chromosome 4, 22% on chromosome 7 and 11% on chromosome 10.

**Fig. 3:**
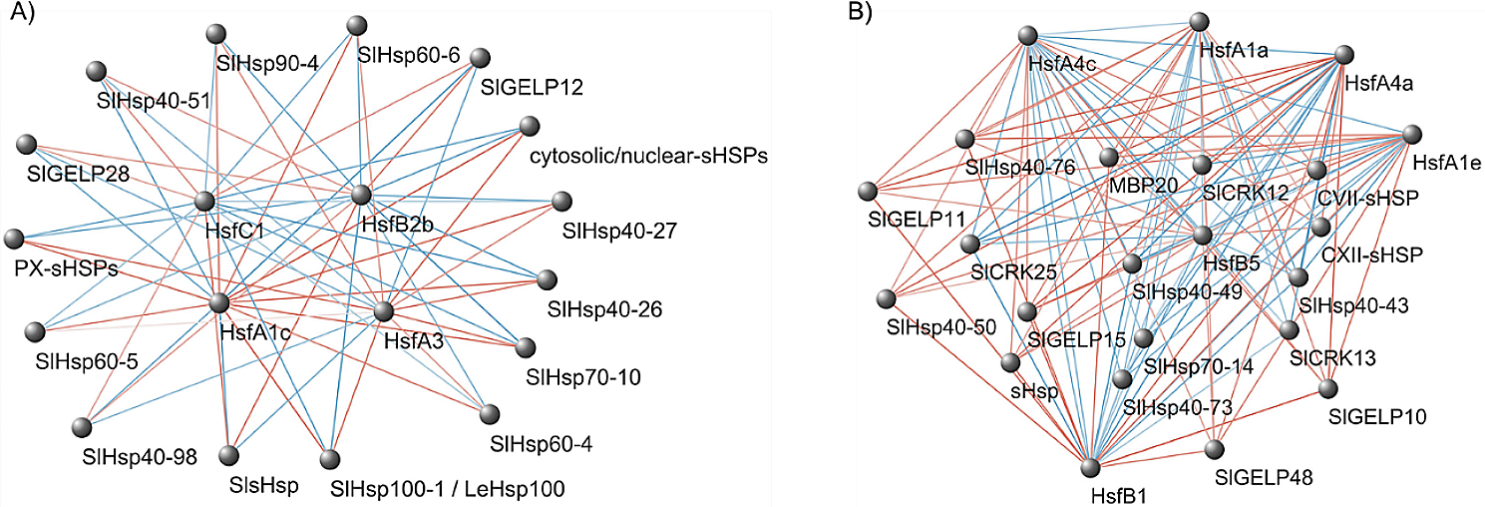
**(A)** Indianred2 and **(B)** honeydew networks showing interactions among heat-related genes containing AGAAnnTTCTRGA and/or CGTTGACY binding motifs in the promoters and TFs for HS response. Red edges refer to negative weight values of interaction, while blue edges refer to positive interactions

Interactions among the highlighted 82 genes and heat-related TFs were investigated in the 52 modules derived from the GCN analysis. Results showed that 21 out of 82 genes did not interact with TFs (Additional file 13). Focusing on the association involving Hsfs, four of these (HsfA1c, HsfA3, HsfB2b and HsfC1) interacted with 15 HS-related genes within the indianred2 module (Fig. 3A), including 13 Hsps and two GELPs, while six Hsfs (HsfA1a, HsfA1e and HsfA4a, HsfA4c, HsfB1 and HsfB5) interacted with 20 HS-related genes within the honeydew module (Fig. 3B), including nine Hsps, four GELPs, three cysteine-rich receptor-like protein kinases (CRKs) and one flower-related gene. Considering all the interactions between TFs and HS-related genes, these were found also in other seven modules, and 61 genes were identified since they carried one HSE motif on the promoter, belonged to one of the 52 modules and interacted with TFs. Most mapped on chromosomes 2 and 4 (40% and 33%, respectively), while the others on chromosomes 7 (17%) and 10 (10%).

In order to reduce the number of genes to be further investigated in the future, we focused on those showing variants highlighted by Graci et al. [17] with HIGH and/or MODERATE impact in the gene coding regions and mapping in the most polymorphic regions in the genome of E42, which derive from the wild species *S. pimpinellifolium*. This analysis allowed to narrow the number of genes to 13 (Table 2), all showing at least one polymorphism determining a MODERATE impact on the protein, whereas only in one case a polymorphism with a predicted HIGH impact was observed.

**Table 2:** List of the 13 heat related genes showing AGAAnnTTCTRGA and/or CGTTGACY binding motifs in the promoters, presenting HIGH and/or MODERATE variants in the gene sequences and also interacting with TFs for HS response. Data regarding impact of variants were retrieved from Graci et al. [17]

Gene ID	Gene name	HIGH impact	MODERATE impact	Module	TFs interaction
Solyc02g088610	SlHsp100-1 / LeHsp100	0	4	indianred2	HsfA1c, HsfA3, HsfB2b, HsfC1
Solyc02g093600	CI-sHSP	1	2	chocolate3	HsfA6b, HsfA2
Solyc04g016000	HsfB3a	0	1	brown1	HsfB3b
Solyc04g016410	SlHsp40-45	0	5	cornsilk	MBF1c
Solyc04g024840	SlGELP27	0	1	lightskyblue2	SlbZIP10
Solyc04g077430	SlHsp40-49	0	2	honeydew	HsfA1a, HafA1e, HsfA4a, HsfA4c, HsfB1, HsfB5
Solyc07g005820	SlHsp70-14	0	1	honeydew	HsfA1a, HafA1e, HsfA4a, HsfA4c, HsfB1, HsfB5
Solyc07g026810	SlHsp40-73	0	2	honeydew	HsfA1a, HafA1e, HsfA4a, HsfA4c, HsfB1, HsfB5
Solyc07g047690	SlHsp40-74	0	3	grey	SlWRKY3
Solyc07g049440	SlGELP48	0	1	honeydew	HsfA1a, HafA1e, HsfA4a, HsfA4c, HsfB1, HsfB5
Solyc07g055710	HsfA4b	0	1	mediumpurple2	SlNAC1
Solyc07g055720	CVII-sHSP	0	1	honeydew	HsfA1a, HafA1e, HsfA4a, HsfA4c, HsfB1, HsfB5
Solyc07g066290	SlHsp40-79	0	1	green4	SlbZIP32, SlbZIP33, HsfA9

**Fig. 4:**
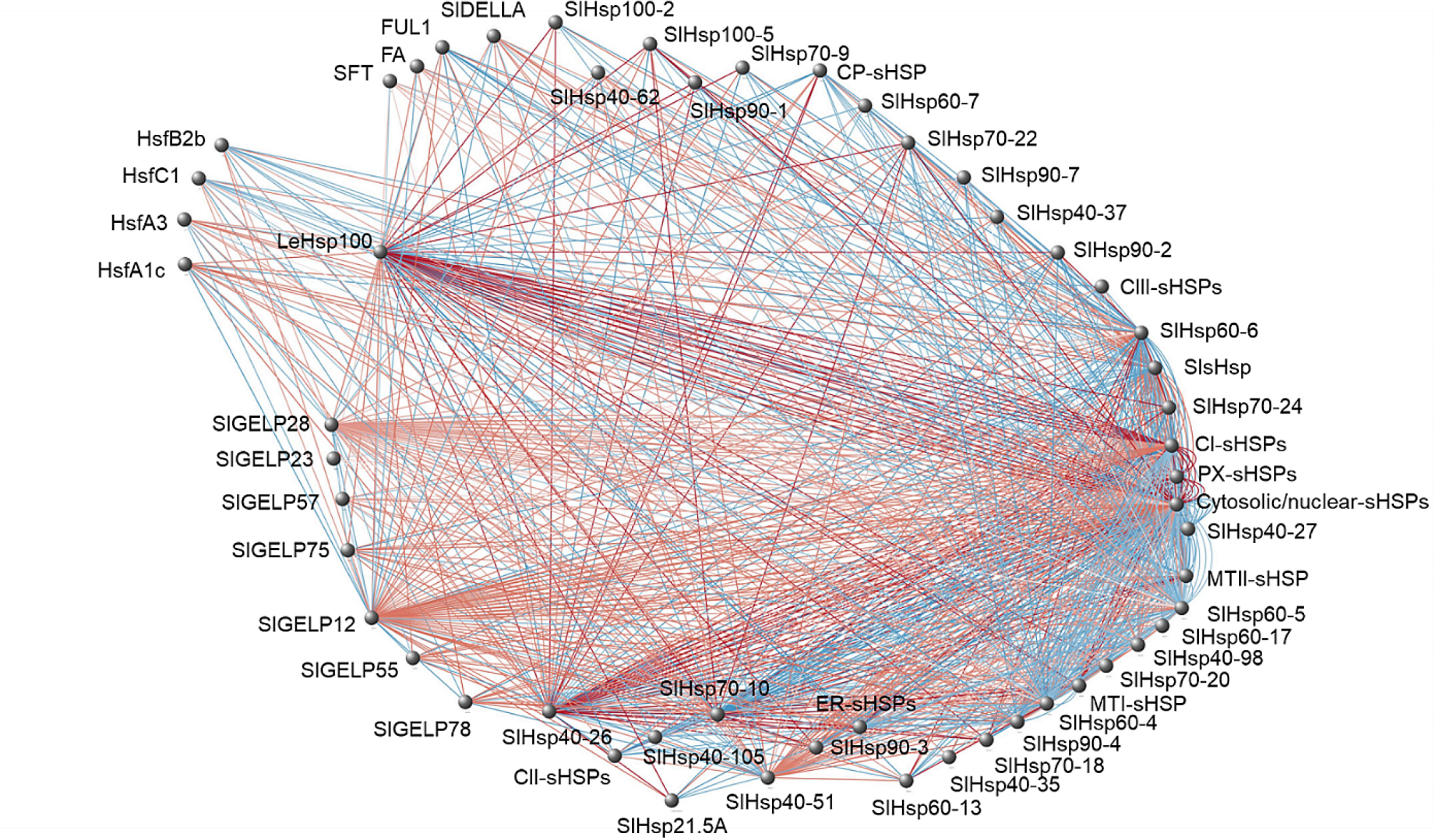
LeHsp100 network showing interactions among heat-related genes. Red edges refer to negative weight values of interaction, while blue edges refer to positive interactions

The list includes two Hsfs (HsfB3a and HsfA4b) presenting one MODERATE mutation and positively interacting with another TF, (HsfB3b for the HsfB3a and SlNAC1 for the HsfA4b); nine Hsps, among which the LeHSP100 that carries four MODERATE mutations, negatively interacts with HsfA1c and HsfA3 and positively interacts with HsfB2b and HsfC1; and two GELPs (SlGELP27 and SlGELP48) with one MODERATE mutation and negatively interacting with their respective TFs. Even though these genes display HSE binding motifs in the promoters and polymorphisms in the gene coding regions, and interacts with TFs, they also interact with a high number of target genes also involved in the HS response. Focusing on the LeHsp100 gene, it belongs to the indianred2 module and presented a complex network that includes four Hsfs, 38 Hsps, three flower-related, seven pollen-related and one fruit set-related genes (Fig. 4).

**Fig. 5:**
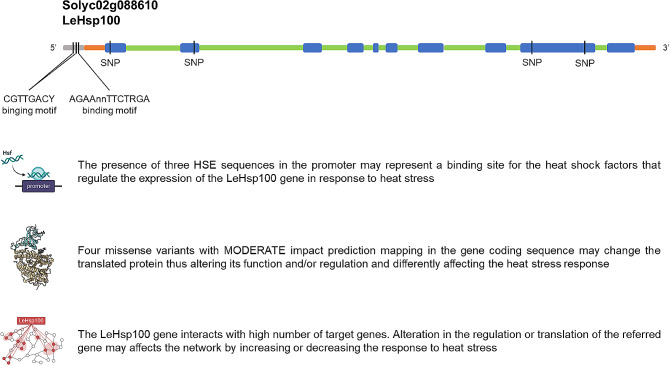
Graphic representation of the genomic features of the LeHsp100 gene in response to high temperatures. Blue and green boxes represent exons and introns, respectively, and orange and grey boxes represent UTRs and promoter regions (Figure modified from Solgenomics, www.solgenomics.net)

The LeHsp100 gene carries one AGAAnnTTCTRGA and two CGTTGACY binding motifs in the promoter and interacts with four Hsfs that could regulate its response under high temperatures. As a whole, in the E42 genotype, this gene showed a series of features that make it eligible as candidate gene in response to high temperatures (Fig. 5).

## Discussion

**Fig. 6:**
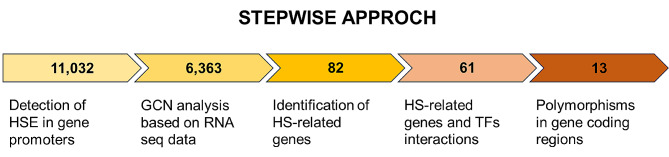
Schematic representation of the five steps of the adopted stepwise approach. The description of procedures and the number of genes highlighted for each of the five steps are reported

Tomato molecular response to HS is orchestrated by a complex network of Hsfs, which play a pivotal role in HS signalling and regulate the expression of several stress-responsive genes [39, 40]. When plants are exposed to high temperatures, the Hsfs, known to be the central regulators of the HS response, regulate the expression of numerous heat shock protein-encoding genes (Hsps) and other targets at the transcriptional level by recognizing conserved binding motifs such as HSEs found in the promoter regions, thus allowing the plant to withstand the stress. These genes are essential in maintaining plant homeostasis under stress conditions and their main functions involve protein folding, unfolding and transport [41–43]. GCN analyses were applied in a high number of studies to identify key genes for specific plant traits. Most of these works focused on transcriptomic analyses of plants of interest performed on a low number of samples or tissues, and allowed to identify key genes and regulatory pathways only on the bases of RNA data [44–46]. In some cases, the authors integrated promoter information of binding motifs from already available databases [47]. In the present work, due to the multiple molecular aspects of thermotolerance, we proposed a stepwise approach exploring genomic and transcriptomic data to reduce the number of genes to be further utilized in the future. This approach will be useful to elucidate the molecular response to high temperature conditions of the heat tolerant E42 genotype, and to identify candidate genes valuable for breeding programs. In this regard, we exploited bioinformatic tools in the following stepwise procedure: (I) detecting the presence of HSE binding sites in the promoter of genes of the genotype; (II) determining the interactions of genes involved in the response to HS; (III) picking up genes related to HS reported in a previous work by Graci et al. [38] among those deriving from steps I and II; (IV) finding those of step III that interact with Hsfs; (V) evidencing the presence of polymorphisms in the gene coding regions of HS-related genes so identified (Fig. 6).

As for the first aspect, in accordance with the findings of Arce et al. [18] and Hichri et al. [19], the AGAAnnTTCTRGA and CGTTGACY binding motifs were identified along the whole E42 genome sequence, focusing on the promoter regions. Results showed that 11,032 genes in the E42 genotype presented the AGAAnnTTCTRGA and/or CGTTGACY motif sequences. Not only the presence or absence of HSEs, but also their number in the promoters could affect the regulation of genes under HS. However, the contribution of the number of the binding sites in the promoter on the regulation of the gene expression remains not fully understood. The second point of our research focused on the interaction of genes [48–50]. In this context, weighted GCN analysis is a bioinformatic application for exploring the relationships between different gene clusters (modules). This method allows to study the correlation patterns between genes and provides straightforward biologically functional interpretations of gene network modules [51]. In order to highlight the HS-related gene interactions and integrate this information with those retrieved from the identification of HSE binding motifs in the promoter, GCN analysis was conducted by using RNA-seq data retrieved from 153 RNA samples extracted from four tomato tissues. The list of genes combining the presence of HSE motifs and belonging to one of the 52 gene networks resulted in 6,363 genes. Moreover, in the third step of our approach, 82 genes related to the HS response according to Graci and Barone [38] were identified from the list of 6,363 genes deriving from the combination of genes obtained from steps I and II. In that previous work, several genes influencing final yield in tomato plants have been identified, such as TFs, Hsps, genes related to flower, flowering, pollen and fruit set, and epigenetic mechanisms involving DNA methylation, histone modification, chromatin remodeling and non-coding RNAs. From the position on tomato chromosomes of the 393 genes so described, some hotspots of genes potentially affecting the response to HS were identified, often co-localizing with QTLs, such as those for stigma exertion, numbers of flowers, numbers of fruits [52–55]. In the fourth step, these 82 genes were investigated for the Hsfs interactions within the network they belong to, and consequently the list of genes was narrowed to 61. Indeed, Hsfs play a key role by detecting stress signaling and regulating the expression of several stress-responsive genes under HS by the binding with HSEs distributed in the promoter regions of the targeted genes [38, 39]. The final aspect of our work aimed at identifying genes with polymorphisms affecting the translated protein, since not only a differential gene expression could affect the mechanism of HS response of the E42 genotype, but also the presence of polymorphisms in the gene coding sequence, which change the amino acid sequence of the protein and probably its function. In a previous work, Graci et al. [17] identified 140 and 54 variants with HIGH and/or MODERATE impact on the translated protein by investigating the coding sequence of 246 heat- and 83 reproductive-related genes, respectively, in the E42 genotype. Among the list of 61 HS-related genes exhibiting the AGAAnnTTCTRGA and/or CGTTGACY motif sequences and belonging to one of the 52 modules, 13 showed variants with HIGH and/or MODERATE impact. These SNP and InDel variations could potentially contribute to altering the protein function thus enhancing or decreasing the HS response of E42. In accordance with these outcomes, Garg et al. [56] found one SNP in the sequence of the HSP16.9 between a heat tolerant and heat susceptible wheat genotypes, resulting in a missense mutation. This SNP contributed 29.89% phenotypic variation for grain weight per spike. The authors provided the first report of HSP-derived SNP marker that can be used for improving tolerance to high temperatures in wheat breeding programs. However, thermotolerance is a quantitative trait that involves a high number of genes, and we should expect that a single molecular marker contributes little to improving its response. Hence, it is crucial to incorporate several SNPs associated to various QTLs involved in the HS response [57–60].

Finally, the combination of the results obtained by investigating the already mentioned five aspects of the present work allowed to extract a list of 13 genes that could be directly involved in the E42 molecular response to HS. All these genes showed the AGAAnnTTCTRGA and/or CGTTGACY binding motifs in the promoter and were found to interact with at least one TF involved in the HS response through the GCN analysis. Basically, these results suggest that TFs could bind the HSE sequences in the promoters of the 13 genes thus regulating their expression, and that at the same time SNP or INDEL variations could also change their protein function. Moreover, alterations in the expression of these genes caused by polymorphisms in the promoters or gene coding sequence may significantly affect the regulation of target genes, thereby influencing the whole network and consequently the HS plant response.

Among the 13 genes highlighted in this work, the LeHsp100 was the only belonging to the indianred2 module, the most enriched in the HS response, and could be the most interesting for future application in breeding programs. Yang et al. [61] provided the first example that the induction of the chloroplast LeHSP100 gene contributes to the acquisition of thermotolerance in tomato plants. Indeed, both transcript and protein LeHSP100 sequences were induced by increasing temperatures while were not detected under normal growth conditions. In addition, Gul et al. [41] also investigated the role of the LeHSP100 gene. They analyzed the expression levels in leaves of five-week-old tomato seedlings following exposure to HS (45 °C) and control (25 °C) and they found that the LeHSP100 gene was upregulated in all the tomato genotypes after the heat treatment, highlighting its key role in acquired thermotolerance. In the E42 genotype, this gene presents one AGAAnnTTCTRGA and two CGTTGACY motifs in the promoter that could be bound by four Hsfs, and four MODERATE polymorphisms in the coding region of the gene that will give a different protein that could modify the HS response. Additionally, it also interacts with several target genes such as Hsps, reproductive-related genes like Single Flower Truss (SFT), Falsiflora (FA), GELPs and PROCERA. Some of these genes also exhibited polymorphisms in the coding regions. Generally, SFT and FA work in parallel pathways to enhance the floral transition of the shoot apical meristem, leading to the repression of the vegetative growth in tomato. In E42, the FA gene showed one missense variant in the gene with MODERATE impact on the protein. *fa* mutants convert many flowers in secondary buds and produce highly branched inflorescences [62, 63]. These mutants are unable to develop complete flowers and have a late flowering phenotype, increasing the number of leaves below the first and successive inflorescences [64]. Moreover, LeHsp100 also interact with other three Hsps (SlHsp40-105, SlHsp70-20 and SlHsp70-24) exhibiting missense variants in the gene coding sequence. Hsp40s are small chaperones mainly involved in a high number of essential cellular processes, including protein folding/unfolding, assembly/disassembly and degradation [65, 66]. On the other hand, the Hsp70 family play a key role in maintaining internal cell stability. Under control conditions, the binding between Hsp70/Hsp90 inhibits the activity of the HsfA1s, while exposure to high temperatures triggers protein deformation/denaturation thus promoting the work of the master regulator [67]. The Hsp70 also acts as molecular chaperon and binds to denatured proteins to restore protein homeostasis inside the cell [67–69]. In addition to Hsfs and Hsps, the list of 13 genes included two GEPLs (SlGELP27 and SlGELP48). This family contains many functional genes playing a crucial role in the regulation of plant growth, morphogenesis of tissues and organs and plant response to stresses [70]. Studies conducted on *Arabidopsis thaliana* have shown that GELPs are also involved in pollen fertility. Tsugama et al. [71] reported that a knockout of one of these genes (GELP77) causes male sterility and failure of pollen separation. Particularly, in the present work we found that the SlGEPL27 interacts with the SlbZIP10 TF. Although its role in tomato thermotolerance is not fully understood, Li et al. [72] studied the expression of 26 tomato bZIPs thus identifying three genes of this family (SlbZIP10, SlbZIP32 and SlbZIP33) that were up-regulated in leaf and root tissues under HS.

## Conclusions

Plants tolerance to high temperatures is a quantitative trait, thus determined by the action of many different genes. In this work, we expanded the knowledge on the molecular response to HS of the thermotolerant E42 genotype, recently published by Graci et al. [17], by investigating the molecular mechanisms through a promoter analysis based on the identification of HSE binding sites on the E42 genome sequence. In addition, a GCN analysis carried out on transcriptomic RNA-seq tomato data highlighted interactions among heat-related genes. These results were combined with those obtained by Graci et al. [17] regarding the presence of variants in the gene coding sequences, thus obtaining a final list of 13 candidate genes involving two Hsfs, nine Hsps and two GELPs. These genes present HSE binding motifs in the promoters, interact with Hsfs and heat-related genes and show variants with HIGH and/or MODERATE impact. Firstly, Hsfs interacting with target genes showing HSE binding sites in the promoters could enhance their regulation and improve the HS response. Moreover, the presence of variants in the gene coding regions may lead to the translation of different proteins that could increase or decrease the thermotolerance by altering their functions and/or the regulation of downstream genes. Finally, target genes could also be affected by an altered regulation or by the presence of polymorphisms changing the protein that may induce changes in the networks thus affecting the response mechanisms. These networks represent an excellent starting point by giving an overall representation of a high number of gene communications potentially occurring in E42 when considering different plant and fruit tissues. In future, the differential expression levels of the selected genes under various HS conditions will be assessed in order to understand their function and validate the molecular response mechanisms of the E42 genotype, also through the application of the genome editing approach.

### Electronic supplementary material

Below is the link to the electronic supplementary material.

Supplementary Material 1

Supplementary Material 2

Supplementary Material 3

Supplementary Material 4

Supplementary Material 5

Supplementary Material 6

Supplementary Material 7

Supplementary Material 8

Supplementary Material 9

Supplementary Material 10

Supplementary Material 11

Supplementary Material 12

Supplementary Material 13

## Data Availability

The datasets analyzed during the current study are publicly available in the NCBI Sequence Read Archive under accession numbers GSE163914, GSE152620, GSE199011 and GSE148217. The E42 resequencing data used and analyzed during the current study are available from the corresponding author on request.

## Abbreviations

BP: Biological Process
CC: Cellular Component
CRK: Cysteine-Rich receptor-like protein Kinases
GBS: Genotyping-By-Sequencing
GCN: Gene Co-expression Network
GELP: GDSL esterase/lipase
GEO: Gene Expression Omnibus
GO: Gene Ontology
HS: Heat Stress
HSE: Heat Stress Element
Hsf: Heat stress transcriptional factor
Hsp: Heat shock protein
InDel: Insertion and/or Deletion
MF: Molecular Function
QTL: Quantitative Trait Loci
PCA: Principal Component Analysis
PWM: Positional Weight Matrix
RNA-seq: RNA sequencing
sHSP: small Heat shock protein
SNP: Single Nucleotide Polymorphism
TF: Transcriptional Factor
VCF: Variant Calling Format
