# Supplementary material for: Exploring the gene expression network involved in the heat stress response of a thermotolerant tomato genotype
Source: BMC Genomics. 2024 May 23;25:509. doi: 10.1186/s12864-024-10393-0 (PMC11112777; doi:10.1186/s12864-024-10393-0)
Supplement: Supplementary file 2 — Supplementary Material 2 [file 12864_2024_10393_MOESM2_ESM.docx]

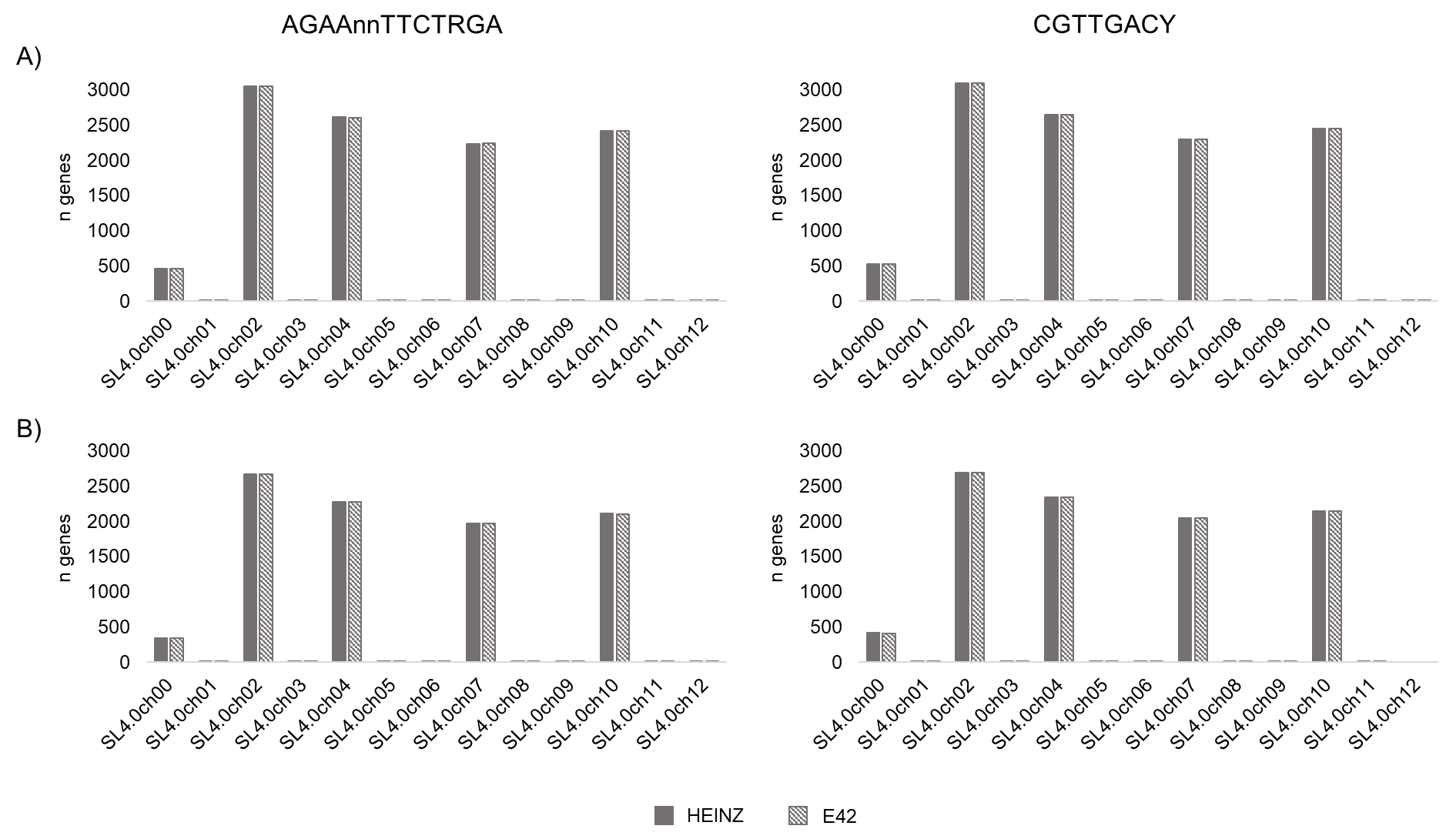


**Additional file 2** - Number of genes per chromosome presenting AGAAnnTTCTRGA and CGTTGACY motifs in the promoters by using A) the intersectbed command of bedtools and B) the ChIPseeker R package of Bioconductor.
