# Supplementary material for: Exploring the gene expression network involved in the heat stress response of a thermotolerant tomato genotype
Source: BMC Genomics. 2024 May 23;25:509. doi: 10.1186/s12864-024-10393-0 (PMC11112777; doi:10.1186/s12864-024-10393-0)
Supplement: Supplementary file 3 — Supplementary Material 3 [file 12864_2024_10393_MOESM3_ESM.docx]

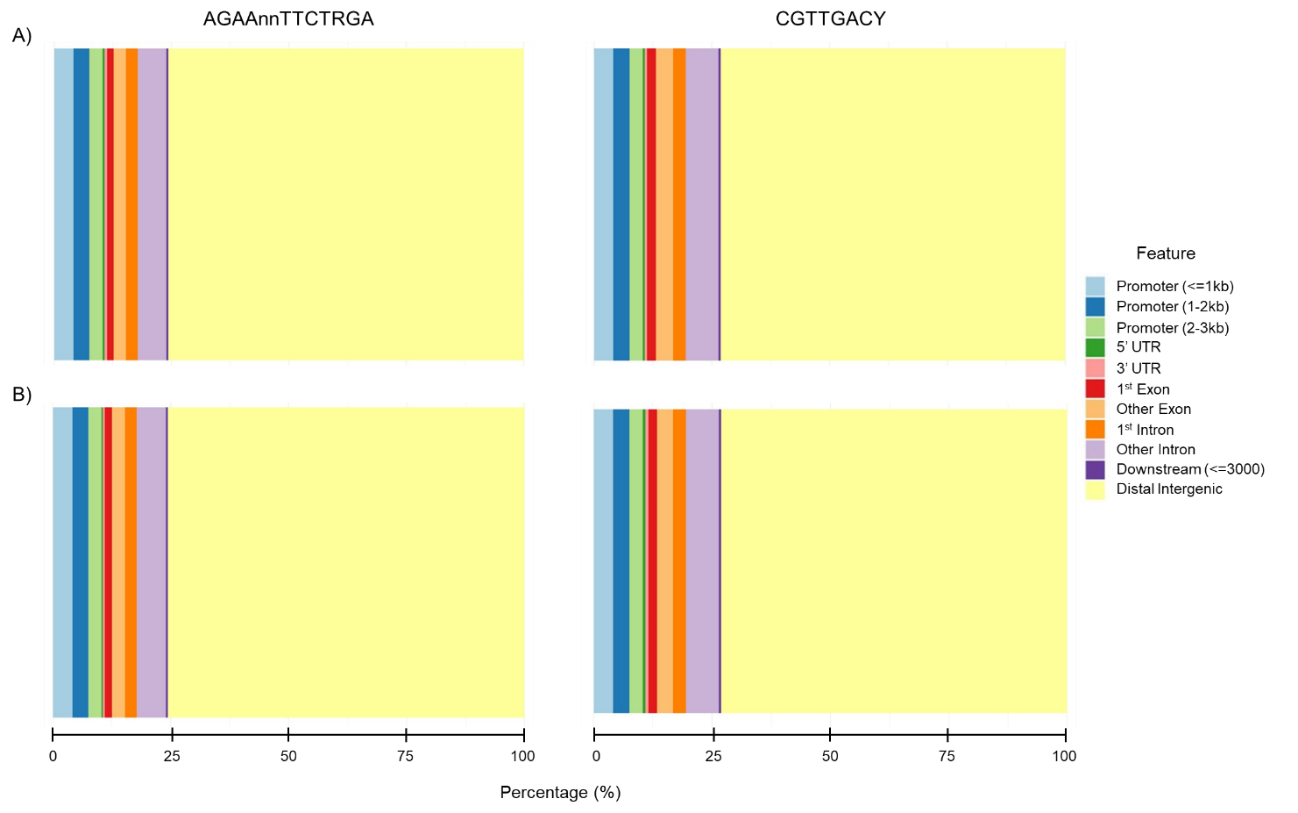


**Additional file 3** - Distribution of AGAAnnTTCTRGA and CGTTGACY binding motif sequences across the A) Heinz and B) E42 genomes.
