# Supplementary material for: Exploring the gene expression network involved in the heat stress response of a thermotolerant tomato genotype
Source: BMC Genomics. 2024 May 23;25:509. doi: 10.1186/s12864-024-10393-0 (PMC11112777; doi:10.1186/s12864-024-10393-0)
Supplement: Supplementary file 6 — Supplementary Material 6 [file 12864_2024_10393_MOESM6_ESM.docx]

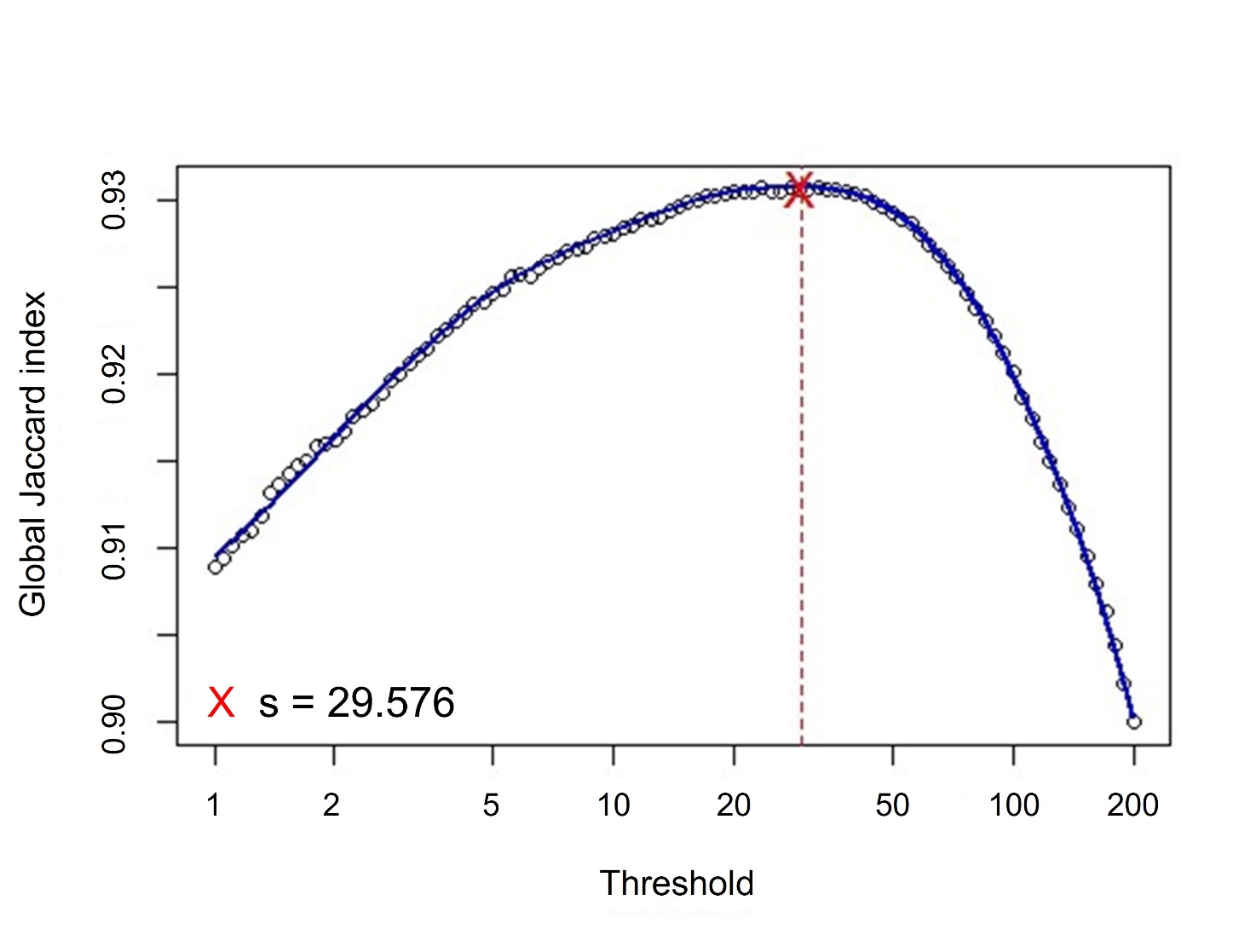


**Additional file 6** - Graphic representation of TMM value at which the Jaccard index is maximum for the normalization of the sequencing data. The index is calculated for a variety of threshold values after TMM normalization, with a loess curve (blue line) superposed and data-based threshold values (red cross and red dotted line) equal to 29.576. Figure was generated by using the HTSFilter function in R.
