# Supplementary material for: Exploring the gene expression network involved in the heat stress response of a thermotolerant tomato genotype
Source: BMC Genomics. 2024 May 23;25:509. doi: 10.1186/s12864-024-10393-0 (PMC11112777; doi:10.1186/s12864-024-10393-0)
Supplement: Supplementary file 7 — Supplementary Material 7 [file 12864_2024_10393_MOESM7_ESM.docx]

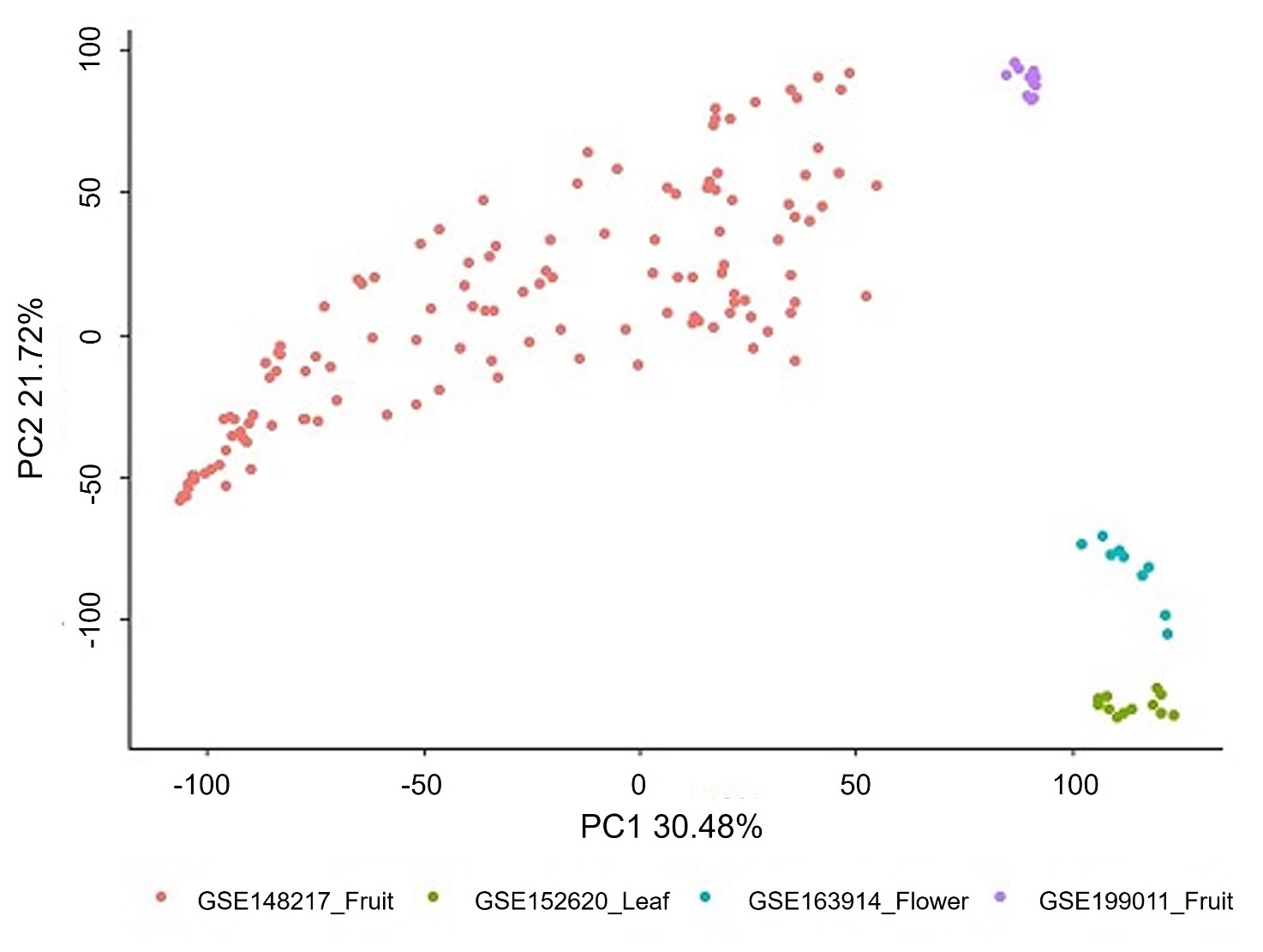


**Additional file 7** - Principal Component Analysis (PCA) conducted on the RNA sequencing data of the 153 tomato samples of the GSE148217, GSE152620, GSE163914 and GSE199011 projects. Colors refer to different tomato tissues: Fruit pericarp and epidermal tissue of the blossom end halves (red), Leaf (green), Flower (blue), Fruit mesocarp (violet). Figure was generated by using the ggplot2 package of R.
