# Supplementary material for: Exploring the gene expression network involved in the heat stress response of a thermotolerant tomato genotype
Source: BMC Genomics. 2024 May 23;25:509. doi: 10.1186/s12864-024-10393-0 (PMC11112777; doi:10.1186/s12864-024-10393-0)
Supplement: Supplementary file 8 — Supplementary Material 8 [file 12864_2024_10393_MOESM8_ESM.docx]

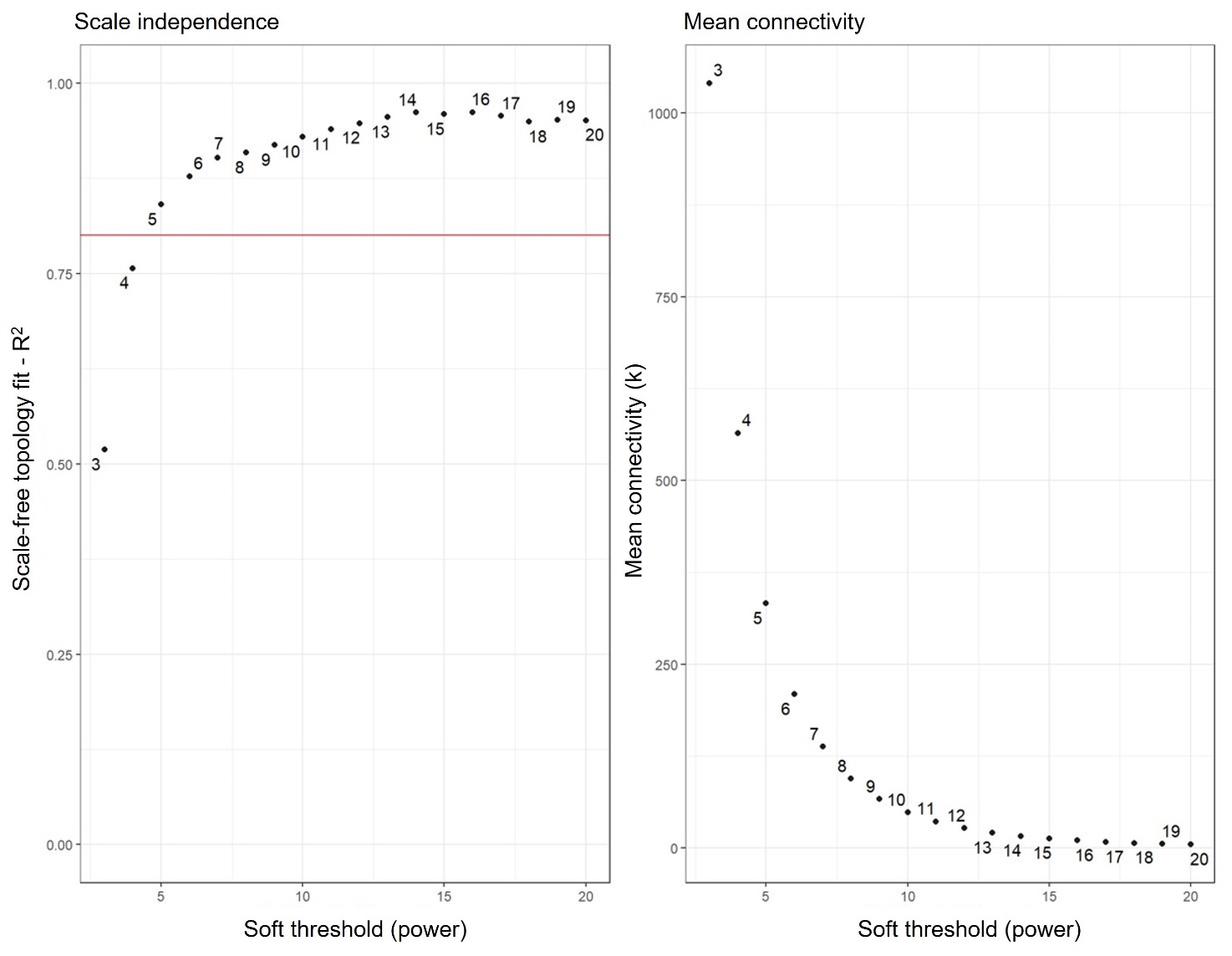


**Additional file 8** - Graphic representation of the selection of the proper soft-threshold power β for WGCNA. The scale-free fit index of network topology was determined by soft-thresholding power analysis. The figure was generated by using the BioNero package of R.
